# Potential Public Health Impact of New Tuberculosis Vaccines

**DOI:** 10.3201/eid1009.030921

**Published:** 2004-09

**Authors:** Elad Ziv, Charles L. Daley, Sally Blower

**Affiliations:** * University of California—San Francisco, San Francisco, California, USA;; † San Francisco General Hospital, San Francisco, California, USA;; ‡ David Geffen School of Medicine at UCLA, Los Angeles California, USA

**Keywords:** mathematical model, tuberculosis, vaccines, health policy, uncertainty analysis, Monte Carlo, perspective

## Abstract

Developing effective tuberculosis (TB) vaccines is a high priority. We use mathematical models to predict the potential public health impact of new TB vaccines in high-incidence countries. We show that preexposure vaccines would be almost twice as effective as postexposure vaccines in reducing the number of new infections. Postexposure vaccines would initially have a substantially greater impact, compared to preexposure vaccines, on reducing the number of new cases of disease. However, the effectiveness of postexposure vaccines would diminish over time, whereas the effectiveness of preexposure vaccines would increase. Thus, after 20 to 30 years, post- or preexposure vaccination campaigns would be almost equally effective in terms of cumulative TB cases prevented. Even widely deployed and highly effective (50%–90% efficacy) pre- or postexposure vaccines would only be able to reduce the number of TB cases by one third. We discuss the health policy implications of our analyses.

## Abstract 2

Widely deployed and highly effective pre- or postexposure vaccines would reduce the number of TB cases by only one third.

Tuberculosis (TB) remains one of the leading causes of illness and death in the world. One third of the world's population is estimated to be infected with *Mycobacterium tuberculosis*, the causative agent of TB (1). This reservoir of infected persons leads to ≈8 million new cases of TB and 2 million deaths each year. Approximately 80% of all new TB cases in the world occur in 22 countries that have incidence rates from 68 to 584 per 100,000 population (2). The priorities for TB control programs in these areas are identifying and treating active cases. Unfortunately, only 40% of smear-positive pulmonary cases are detected globally, and, of these cases, 28% to 80% are treated successfully (2). Most high-incidence countries also use the only available TB vaccine, *Mycobacterium bovis* bacillus Calmette-Guérin (BCG). Although BCG is the most widely used vaccine in the world, its efficacy in preventing adult forms of TB is relatively poor, with an average efficacy ≈50% (3). A new, more effective vaccine would be expected to improve TB control substantially, and therefore, vaccine development is one of the highest priorities in TB research (4,5). The Gates Foundation recently provided nearly $83 million in grants to boost TB vaccine research (6).

Recent sequencing of the *M. tuberculosis* genome as well as new developments in proteomics and comparative genomics have led to renewed interest in developing new, more effective vaccines against TB (7,8). Vaccines currently under development include subunit vaccines (9), naked DNA vaccines (10,11), and attenuated mycobacteria, including recombinant BCGs expressing immunodominant antigens and cytokines (12). Phase I clinical trials of several of these vaccines are under way or scheduled to begin very soon (13,14). TB vaccines under development can be divided into two categories: preexposure or postexposure vaccines. Preexposure vaccines prevent infection and subsequent disease; these vaccines are given to uninfected persons. Postexposure vaccines aim to prevent or reduce progression to disease; these TB vaccines will be given to persons who are already infected with *M. tuberculosis*. In industrialized countries where TB incidence is low, a preexposure vaccine is the most effective for TB control (15). However, the most effective type of vaccine to control TB epidemics in high-incidence countries, where prevalence of latent TB infection is high, is not apparent. We use mathematical models to predict the potential public health effect of new TB vaccines for epidemic control in high-incidence countries. We evaluate the effect of both pre- and postexposure TB vaccines on two outcome variables: the number of new infections and the number of new cases of disease. We then discuss health policy implications of our analyses.

## Prediction Methods

We used mathematical models to compare the potential public health impact of mass vaccination campaigns that used either pre- or postexposure vaccines. We assessed the public health impact in terms of the cumulative percentage of infections prevented and the cumulative percentage of TB cases prevented. We modeled the potential effect of vaccines in developing countries with a high incidence and prevalence of infection. Our simulated incidence ranged from 100 to 200 new TB cases per 100,000 persons per year, and we assumed that 28%–50% of the population was latently infected with *M. tuberculosis*. We also assumed that treatment rates were low to moderate (i.e., that 40%–60% of TB patients would be treated and cured). We modeled the potential public health impact of high-efficacy (50%–90%) vaccines and high vaccination coverage rates (60%–90%). We used two separate mathematical models to assess the effect of vaccination: a pre- and a postexposure vaccine model (see Appendix). Our models are similar to those developed by Lietman and Blower (15,16), but we extended them to include the possibility of reinfection of latently infected persons. We analyzed both of our models with uncertainty and sensitivity analysis based on Monte Carlo methods (17–20) (see Appendix for further details) to quantify the effect of vaccine efficacy, duration of vaccine-induced immunity, and vaccination coverage rates on the cumulative percentage of infections and TB cases prevented.

**Figure 1:**
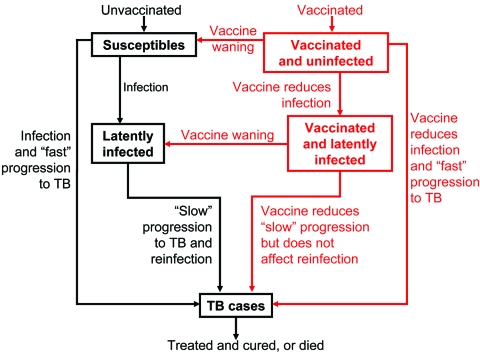
Flow-diagram of postexposure tuberculosis (TB) vaccine model. States and processes that relate to the vaccine are shown in red. Equations are given in the Appendix.

**Figure 2:**
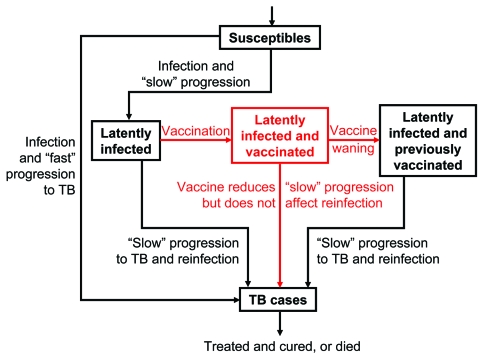
Flow-diagram of preexposure tuberculosis (TB) vaccine model. States and processes that relate to the vaccine are shown in red. Equations are given in the Appendix.

Both our vaccine models reflect the basic pathogenesis of TB (Figures 1 and 2), as in our previous models (21–27). When persons become infected with *M. tuberculosis*, one of the following can occur: 1) they can progress quickly to disease (with probability *p*); 2) they can become latently infected with *M. tuberculosis* (with probability 1 – *p*), and disease never develops; or 3) they can become latently infected with *M. tuberculosis* (with probability 1 – *p*) and slowly progress to disease (at rate *v*). Latently infected persons can also become reinfected (with a relative risk of *θ*) with a new strain of *M. tuberculosis*. We assessed the potential public health impact of 1,000 different postexposure and 1,000 different preexposure vaccines. Each vaccine had a different efficacy (50%–90%) and average duration of vaccine-induced immunity (10–30 years). We modeled vaccination coverage rates from 60% to 90%. We modeled a mass vaccination campaign at year zero, and then continuous vaccination of each target population each subsequent year.

Our pre- and postexposure vaccine models were designed to vaccinate different populations: preexposure vaccines were designed for uninfected persons, and postexposure vaccines were designed for latently infected persons. We modeled vaccine efficacy for the 1,000 postexposure vaccines by the magnitude of the vaccine's effect on reducing the rate of latently infected persons' progressing to disease (Figure 1). Efficacy of preexposure vaccines is potentially more complex than that of postexposure vaccines, since preexposure vaccines have several potential mechanisms of action. Thus, we assumed that preexposure vaccines could act by three different mechanisms (Figure 2): 1) by reducing the risk for infection in the uninfected, 2) by allowing infection but reducing the probability of fast progression to disease, and 3) by allowing infection but reducing the rate of progression of latent infection to clinical disease. For each of our 1,000 preexposure vaccines, we varied these three potential mechanisms independently from 50% to 90%.

## Percentage of Infections and Cases Prevented

**Figure 3:**
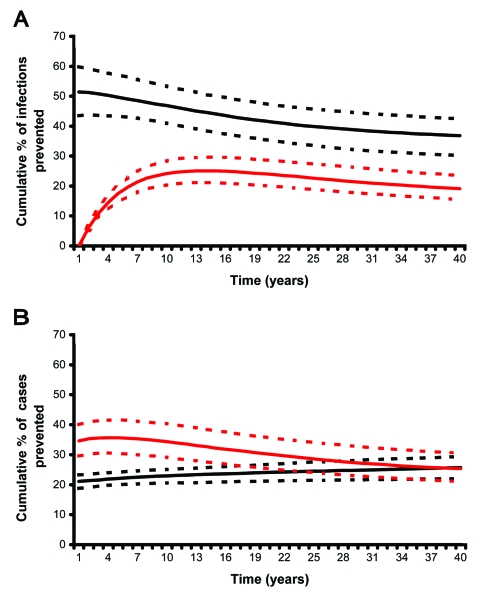
A) Cumulative percentage of new infections with Mycobacterium tuberculosis prevented. B) Cumulative percentage of tuberculosis cases prevented. Predictions made using either the preexposure (black lines) or postexposure (red lines) vaccine models and uncertainty analysis.

In terms of reducing the cumulative number of new infections with *M. tuberculosis*, we found that campaigns that used preexposure vaccines had substantially greater effectiveness than campaigns that used postexposure vaccines (Figure 3A). Preexposure vaccines quickly and substantially reduced the number of new infections; the median cumulative percentage of infections prevented (after 10 years of vaccination) was 46% (interquartile range [IQR] 40%–53%). The effectiveness of preexposure vaccines in preventing new infections diminished over several decades but remained fairly high. Postexposure vaccines had a considerably slower and smaller effect on reducing the number of new infections; the cumulative percentage of infections prevented rose from 0% (when mass vaccination began) and peaked after ≈10 years at a median of 25% (IQR 21%–29%) (Figure 3A). After 10 years, the effectiveness of postexposure vaccines in preventing new infections gradually declined.

In contrast, in terms of reducing the cumulative number of new cases of TB, postexposure vaccines initially had substantially greater effectiveness than preexposure vaccines. After 10 years of vaccination, postexposure vaccines had reduced the cumulative number of TB cases by a median of 34% (IQR 29%–40%) (Figure 3B); effectiveness diminished slightly over the next few decades, despite continuous vaccination of newly infected latent persons (Figure 3B). Preexposure vaccines, despite having reduced the infection rate by 46% (IQR 40%–53%) (Figure 3A), only reduced the cumulative percentage of TB cases by a median of 23% (IQR 21%–25%) after 10 years (Figure 3B). After 20 to 30 years of continuous vaccination, post- and preexposure vaccines had similar effectiveness in terms of the cumulative percent of TB cases prevented (Figure 3B).

## Coverage Rates, Duration of Immunity, and Vaccine Efficacy

**Figure 4:**
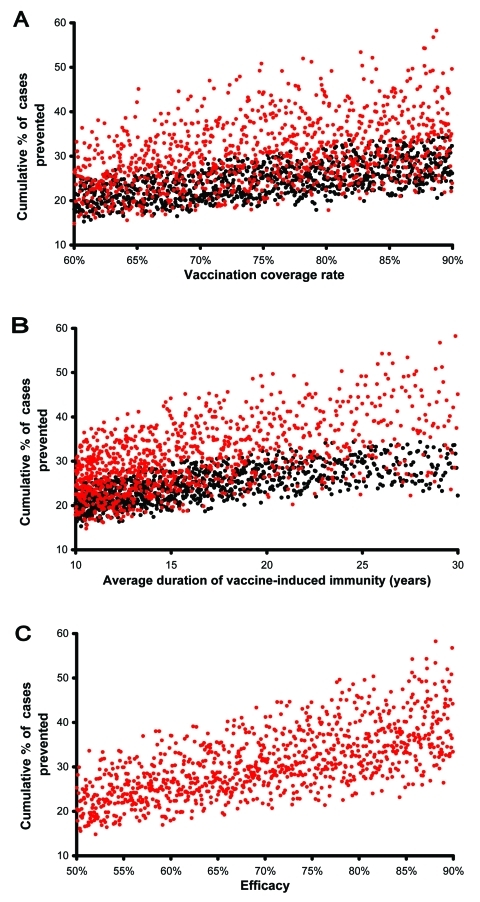
Unadjusted predicted data are plotted; red points represent postexposure vaccines, black points represent preexposure vaccines. A) Cumulative percentage of tuberculosis (TB) cases prevented. B) Cumulative percentage of TB cases prevented. C) Cumulative percentage of TB cases prevented. Cases prevented after 20 years of vaccination are shown as a function of vaccination coverage rates, duration of vaccine-induced immunity, or vaccine efficacy.

To predict the potential public health impact of pre- and postexposure vaccines, in our uncertainty analysis we varied vaccination coverage rates, duration of vaccine-induced immunity, and vaccine efficacy. We determined the quantitative effect of each of these three variables on the cumulative percentage of TB cases prevented by performing a multivariate sensitivity analysis and calculating partial rank correlation coefficients (PRCCs) (Appendix). The cumulative percentage of TB cases prevented increased substantially (PRCC = 0.93, 0.96) as vaccination coverage rates increased from 60% to 90% (Figure 4A, unadjusted data after 20 years of continuous vaccination); this effect was greater for postexposure vaccines than preexposure vaccines. The cumulative percentage of TB cases prevented also increased substantially (PRCC = 0.95, 0.97) as the average duration of vaccine-induced immunity increased from 10 to 30 years (Figure 4B, unadjusted data after 20 years of continuous vaccination); this effect was greater for postexposure vaccines than preexposure vaccines.

We assessed the effectiveness of 1,000 postexposure vaccines that varied in efficacy from 50% to 90%; vaccine efficacy was defined by the degree of reduction in the disease progression rate of latently infected persons. The cumulative percentage of TB cases prevented increased substantially as the postexposure vaccine efficacy increased from 50% to 90% (Figure 4C, unadjusted data after 20 years of continuous vaccination, PRCC = 0.97). Efficacy of preexposure vaccines is more complex than that of postexposure vaccines; therefore, we modeled the efficacy of preexposure vaccines by three different mechanisms (Figure 2) and evaluated the effect of each of the three mechanisms on the cumulative percentage of TB cases prevented. We assumed that preexposure vaccines could reduce the risk for infection in the uninfected (mechanism 1), allow infection but reduce the probability of rapidly progressing to disease (mechanism 2), and allow infection but reduce the rate of progression of latently infections to disease (mechanism 3). We varied each of these three potential mechanisms independently to vary efficacy levels from 50% to 90%. Preexposure vaccines that operated by using mechanism 3 were not effective (PRCC < 0.5) at preventing a substantial cumulative percentage of TB cases, even if these type of preexposure vaccines had a high efficacy. Preexposure vaccines that operated by either mechanism 1 or 2 were effective in preventing TB cases; with preexposure vaccines that operated by reducing the risk of infection in the uninfected (i.e., mechanism 1) being more effective (PRCC = 0.84) than vaccines that operated by allowing infection but reducing the probability of fast progression to disease (i.e., mechanism 2) (PRCC = 0.66).

## Public Health Policy Implications

We evaluated the potential effectiveness of a variety of pre- and postexposure vaccines in controlling TB epidemics in countries that have both a high incidence of disease and a high prevalence of infection. Under these epidemiologic conditions, we found that preexposure vaccines would be almost twice as effective as postexposure vaccines in reducing the infection rate. In contrast, vaccination campaigns that used postexposure vaccines would initially have a substantially greater effect reducing the number of TB cases than campaigns that used preexposure vaccines. However, our predictions show that (despite continuous vaccination) the effectiveness of campaigns using postexposure vaccines would diminish over time but that the effectiveness of campaigns using preexposure vaccines would increase. Hence, after 20 to 30 years, campaigns using either postexposure or preexposure vaccines would be equally effective (because of the complexity of the vaccine mechanisms that we modeled) in terms of the cumulative number of TB cases prevented.

Since preventing disease is more important than preventing infection and to have an immediate, substantial decrease in TB cases is desirable, our results imply that postexposure vaccines would be more beneficial than preexposure vaccines. Our results show that public health officials should expect campaigns that use postexposure vaccines to first appear highly effective, but that effectiveness will decrease with time. We have also shown that the incidence of disease is likely to remain high even if highly effective vaccines that induce long-term immunity are developed and widely deployed. We found that even widely deployed high-efficacy (50%–90%) pre- or postexposure vaccines are only likely to reduce the number of TB cases by one third. Reductions in the number of TB cases would directly translate into reductions in TB deaths (results not shown). Currently the annual TB death rate is 2 million; hence, our results indicate that the type of vaccines we modeled could save ≈700,000 lives per year. These vaccines could also substantially reduce the emergence of drug-resistant TB (22,24).

To understand why even high-efficacy (50%–90%) vaccines are only capable of reducing the TB death rate by one third, how the natural history of *M. tuberculosis* infection differs from other, more "simple," pathogens (e.g., influenza, measles, and smallpox) needs to be examined. For "simple" pathogens, preexposure vaccines can be very effective in reducing epidemic severity because the incidence of disease is a direct function of the incidence of infection. For a "simple" pathogen, if a vaccine reduces infection rates by 80%, then the vaccine will also reduce disease rates by 80%. However, the natural history TB is more complex: the incidence of disease does not directly reflect the incidence of infection with *M. tuberculosis*. The incidence of disease is driven by two sources: susceptible persons who become infected and quickly progress to disease (source 1) and latently infected persons who slowly progress to disease, often many years after the initial infection (source 2). Both sources make a substantial contribution to the incidence of disease. Preexposure vaccines (given to uninfected persons) will act mainly on reducing the contribution of source 1 to the incidence, but they will have little direct effect on reducing the contribution of source 2. In contrast, postexposure vaccines (given to latently infected persons) will act mainly on reducing the contribution of source 2 to the incidence but will have relatively little effect reducing the contribution of source 1. Therefore, even if highly effective pre- or postexposure vaccines are widely deployed, the incidence of TB in developing countries (as our results show) is likely to remain high.

Also, the increasing HIV epidemic will lead to continuous increases in the incidence of TB in developing countries (26). Currently, what effect co-infection with HIV will have on TB vaccine effectiveness is unclear; possibly, HIV co-infection could reduce vaccine effectiveness. Thus, any new TB vaccine should be evaluated in clinical trials to determine the effect of HIV coinfection on vaccine effectiveness. To reduce the severity of TB epidemics, we recommend that developing and deploying vaccines that act as both pre- and postexposure vaccines are necessary to simultaneously attack both sources that drive the TB rate. Additionally, maintaining high rates of detection and treatment of tuberculosis is necessary, as recommended by the World Health Organization (2); by combining treatment and vaccination strategies, eradicating TB epidemics may be possible, as we have previously shown (16).

Our results have implications for designing both TB vaccines and vaccination campaigns. Highly effective vaccines will be needed to have the public health impact that we have shown (i.e., to reduce the TB death rate by one third). Whether or not the vaccines currently in development will afford this level of efficacy remains to be seen. Moreover, vaccines will need to provide very long-lasting immunity; our current analysis examines the effect of fairly long-lasting vaccines (10–30 years average duration of immunity). Different types of vaccines have different durations of immunity. For example, DNA vaccines should provide lifelong immunity, whereas subunit vaccines will likely require booster vaccinations (28), an approach that would be more logistically difficult and expensive. Also, we have shown that preexposure vaccines are best if they prevent infection (mechanism 1) rather than allow infection but reduce the probability of fast progression to disease (mechanism 2) or reduce the rate of progression of latently infections to disease (mechanism 3). Whether or not new TB vaccines will prevent infection from occurring is not known, but BCG is clearly not able to prevent infection, and vaccines currently in development will likely not be able to do so either (29). As new TB vaccines and other control strategies become available, their potential benefits to TB control efforts can be evaluated by mathematical modeling. Mathematical models can be used as health policy tools to evaluate strategies for controlling TB (30–35); mathematical models also provide insights for predicting the potential public health impact of imperfect HIV vaccines (36–39). Our results show that, because of the complex pathogenic process of TB, high-incidence epidemics are unlikely to be substantially reduced by widely deploying highly effective preexposure or postexposure vaccines. We suggest that to achieve global control of TB, developing a single TB vaccine that functions as both a pre- and a postexposure vaccine is necessary.

## Appendix

### Vaccine Models

#### Preexposure Model

Our preexposure vaccine model consists of six ordinary differential equations (1–6) that track the temporal dynamics of persons in six different states: uninfected unvaccinated (*X*), vaccinated uninfected (*X_v_*), unvaccinated latently infected (*L*), vaccinated latently infected (*L_v_*), active tuberculosis (TB) (*T*), and treated and cured (*R*). The model is given below:

(1) d*X*/d*t* = (1 – c)π – b*XT* – µ*X* + ω*X_v_*

(2) d*X_ν_*/d*t* = *c*π – ε_1_β*XT* – (µ + ω)*X_v_*

(3) d*L*/d*t* = (1 – *p*)β*X_ν_T* – (ν + µ + θ*p*β*T*)*L* + ω*L_v_*

(4) d*L_v_*/d*t* = (1 – ε_2_*p*) ε_1_β*XT* – (ε_3_ν + µ + θ*p*β*T* + ω)*L_&nu:_*

(5) d*T*/d*t* = *p*β*XT* + ε_1_ε_2_*p* βX_ν_T + ω*L* + ε_3_ω*L_ν_* + ω*p*β*T*(*L* + *L_ν_*) – (µ + µ*_T_* + φ)*T*

(6) d*R*/d*t* = φ*T* – µ*R*

Persons enter the population at rate π and a fraction *c* of them are vaccinated. Uninfected-unvaccinated persons (*X*) are infected at rate β*T*(*t*), and then either progress to active disease (*T*) immediately after infection with probability *p*, or progress to latent infection with probability 1 – *p*. Latently infected persons (*L*) progress to active disease because of reactivation of latent infection at rate ν. In addition, latently infected persons (*L*) can also be reinfected at a rate β*T*(*t*) and progress to active disease with probability *p* (the probability of rapid progression for newly infected persons) multiplied by the protection afforded by prior infection from rapid progression (θ). Uninfected vaccinated persons (*X_v_*) are protected from infection by probability ε_1_. Vaccinated persons who become infected (*L_ν_*) are protected from rapid progression to active disease by probability ε_2_. We assume that the vaccine may offer some protection from reactivation (ε_3_). The average duration of vaccine-induced immunity is 1/ω. The average life expectancy is 1/µ. Persons with active TB either die at a rate µ*_T_* or receive effective treatment at a rate φ, which leads to recovery (*R*).

#### Postexposure Vaccine Model

Our postexposure vaccine consists of six ordinary differential equations (7–12) that track the temporal dynamics of persons in six different states: uninfected (*X*), unvaccinated latently infected (*L*), vaccinated latently infected (*L_V_*), previously vaccinated latently infected who have lost immunity (*L_W_*), active disease (*T*), and treated and recovered (*R*). The model is given below:

(7) d*X*/d*t* = (1 – *c*)π – β*XT* – µ*X*

(8) d*L*/d*t* = (1 – *p*) β*XT* – (ν + µ + θ*p*β*T* + χ)*L*

(9) d*L_v_*/d*t* = χ*L* – (εν + µ + θ*p*β*T* + ω)*L*

(10) d*L_w_*/d*t* = ω*L_v_* – (ν + µ + θ*p*β*T*)*L_w_*

(11) d*T*/d*t* = *p*β*XT* + ω(*L* + *L_w_*) + ε_3_ω*L_ν_* + θ*p*β*T*(*L* + *L_ν_* + *L_w_*) – (µ + µ*_T_* + φ)*T*

(12) d*R*/d*t* = φ*T* – µ*R*

Persons enter the population at rate π. They become infected at rate β*T*(*t*) and then either progress rapidly to active disease with probability *p* or progress to latent infection (*L*) with probability 1 – *p*. Latently infected persons (*L*) may progress to active disease at rate ν or become reinfected at rate θ*p*β*T*, where θ defines the protection from reinfection because of natural immunity. Latently infected persons may also be vaccinated. The rate of vaccination is set so that the fraction of latently infected persons who have been vaccinated is equal to *c*. Latently infected persons who have lost immunity have the same probability of reactivation and disease from new infection as uninfected persons. The average life expectancy is 1/µ. Persons with active TB either die at rate µ*_T_* or receive effective treatment at a rate φ, which leads to recovery.

### Uncertainty and Sensitivity Analysis

We analyzed the two vaccine models by using time-dependent uncertainty analysis (40-45) and numerically simulated the models to calculate the cumulative reduction in new infections with *Mycobacterium tuberculosis* and cases of TB. The reduction in new infections and in cases of TB was calculated as the percentage of the cumulative number of new infections or new cases of TB that would have occurred without vaccination (but with treatment). We used probability density functions to specify each parameter in the two models. We then used Latin hypercube sampling, a modified Monte Carlo sampling procedure, to sample all of the probability density functions (ranges are given in the text). To conduct the uncertainty analyses (for each model), we performed 1,000 simulations; full details of the uncertainty analysis methods are given elsewhere (40-47). We modeled the effects of an initial mass vaccination campaign of the target population and then continued vaccinating the target population. To quantify the sensitivity of the outcome variables to each parameter, we calculated a partial rank correlation coefficient between each parameter value and each outcome variable (40-47).

### Parameter Estimates

Our biological parameter values for TB were chosen to simulate epidemics in a high-incidence, high-prevalence region. Estimates for µ, *p*, and µ*_t_*, are previously described (5). We assume that endogenous immunity to disease from reinfection reduces rapid progression from reinfection by 50% to 100%; if protection is 100%, reinfection does not occur.
